# ANA Negative Systemic Lupus Erythematosus Leading to CTEPH, TTP-Like Thrombocytopenia, and Skin Ulcers

**DOI:** 10.1155/2016/4507247

**Published:** 2016-02-24

**Authors:** Khalid Hamid Changal, Fayaz Sofi, Sheikh Shoaib Altaf, Adnan Raina, Ab. Hameed Raina

**Affiliations:** ^1^Internal Medicine, Sher-I-Kashmir Institute of Medical Sciences, Srinagar 190011, India; ^2^Rheumatology and Internal Medicine, Sher-I-Kashmir Institute of Medical Sciences, Srinagar 190011, India; ^3^Internal Medicine, Mercy Catholic Medical Center, Philadelphia, PA 19026, USA

## Abstract

SLE affects almost every organ system, with differing degrees of severity. During its clinical course periods of flares may alternate with periods of remission culminating in disease and therapy related damage. We describe a case of ANA negative SLE with severe thrombocytopenia, cutaneous vasculitis, antiphospholipid antibody syndrome, and pulmonary artery hypertension. As there is no definitive cure for SLE the treatment lies in caring for the individual organ systems involved and simultaneously taking care of the patient as a whole.

## 1. Introduction

Systemic lupus erythematosus (SLE) is an autoimmune disorder that affects multiple organs. ANA (antinuclear antibody) negative SLE is a rare entity. The presentation in this case demonstrates how many organ systems can be affected in SLE and how complex the management is. Also it demonstrates that true ANA negative SLE does exist.

## 2. Case Presentation

**Figure 1:**
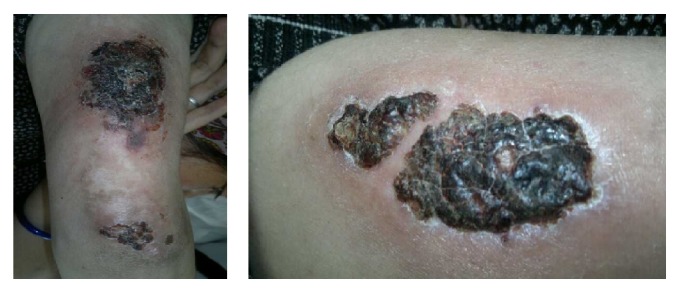
Pictures of the ulcers on the arms of the patient. Crustations have developed on the ulcers.

**Figure 2:**
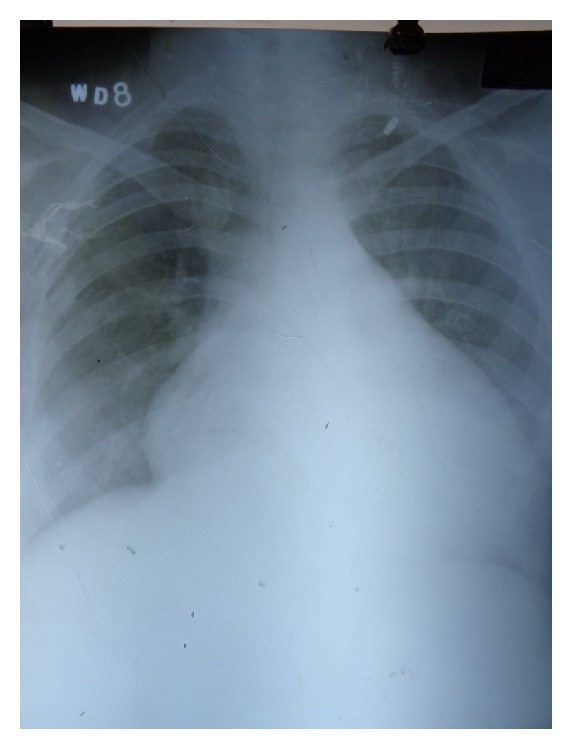
Chest X-ray showing increased cardiothoracic ratio and prominent left pulmonary conus.

**Figure 3:**
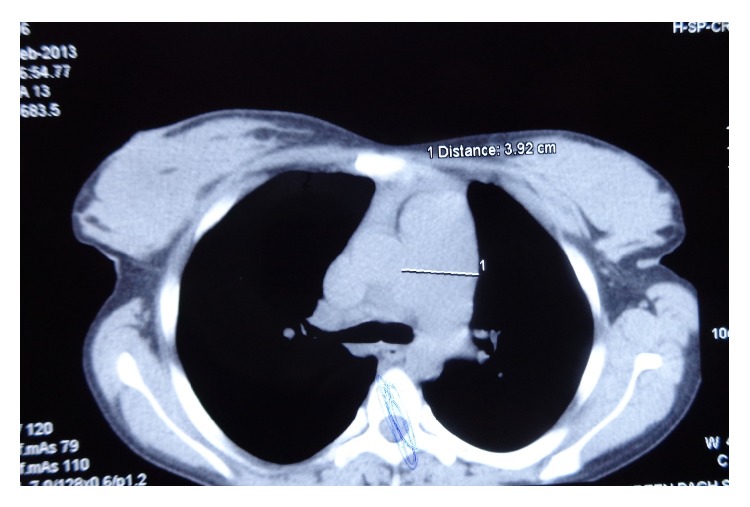
CT chest showing dilated pulmonary artery (marked by 1).

**Figure 4:**
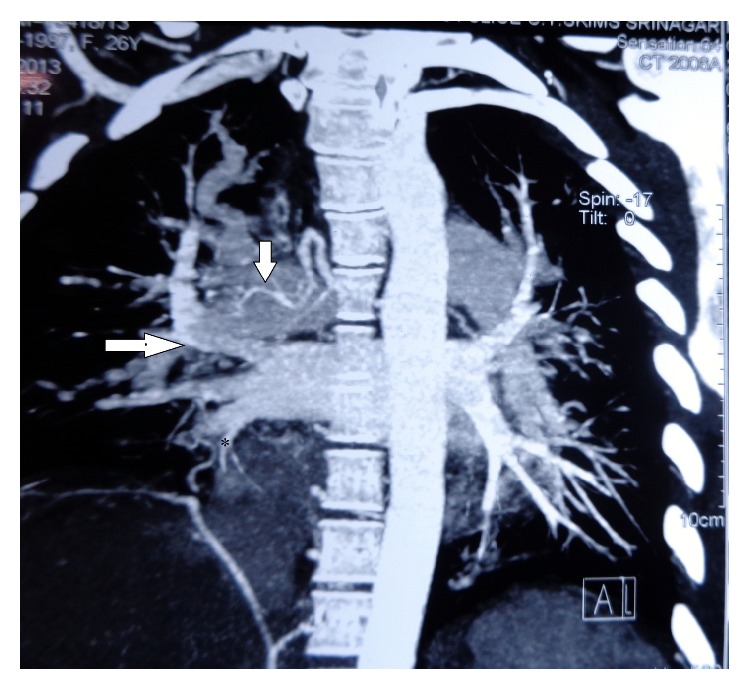
CT pulmonary angiography showing filling defects suggestive of thrombi in the pulmonary vessels (horizontal arrow). Pruning of pulmonary vessels is seen (asterisk). Also collateral vessels have started forming (vertical arrow).

The patient was a 26-year-old woman who had history of recurrent admissions for multiple problems. Despite being admitted multiple times she had not received a diagnosis for her recurrent admissions. Her first presentation was 2 years priorly. She had a right popliteal vein thrombosis for which she was put on treatment with warfarin. 5 months later, she suffered a massive pulmonary thromboembolism despite being on anticoagulation. Bed-side echocardiography had shown positive Mc Connell sign and she was thrombolysed with streptokinase. Warfarin was continued on discharge. Few months later she had an episode of hematemesis due to warfarin induced coagulopathy. Warfarin dose was adjusted. Few months later she was admitted again, this time with skin ulcers on posterior aspect of upper arms which were nonhealing. The current admission was for recurrent skin ulcers (Figure 1) and breathlessness on exertion (functional classes II-III). In addition patient had generalized fatigue, malar rash, and photosensitivity. Hospital course was complicated with hematemesis. Esophagogastroduodenoscopy showed shallow erosions in stomach. Cardiovascular examination showed signs of right ventricular hypertrophy. A thorough evaluation was started. Hemogram showed anemia and thrombocytopenia. Platelet count was 20,000/micl. Peripheral blood film showed schistocytes and her serum lactate dehydrogenase was high. C-reactive protein was high. C3 levels were low. Other biochemical investigations were normal. Urinalysis was normal and there was no proteinuria. Chest X-ray showed increased cardiothoracic ratio and prominent left pulmonary conus (Figure 2). ANA and dsDNA were negative. ANA was tested using indirect immunofluorescence (IF-ANA) using HEp-2 cell substrates. dsDNA was tested using IF-ANA test using* Crithidia luciliae* as the substrate. Doppler ultrasound of lower limbs showed no evidence of DVT. An ultrasonogram of the abdomen showed congestive hepatosplenomegaly and mild ascites. Echocardiography showed normal left heart valves and function. Pulmonary artery was dilated. There were signs of severe pulmonary artery hypertension and severe tricuspid regurgitation. Investigations for APLA (antiphospholipid antibody) syndrome were ordered. Anti-Beta 2 glycoprotein antibody (anti *β*-2 gp Ab), lupus anticoagulant (LA), and anti-cardiolipin antibodies (ACL) were present. HRCT (high resolution computed tomography) of chest showed normal lung parenchyma but right ventricular and right atrial enlargement with signs of pulmonary artery hypertension (Figure 3). A CT pulmonary angiogram was done to evaluate the vasculature of lungs. The main pulmonary artery was dilated with a diameter of 3.6 cm. There were few filling defects in subsegmental branches of pulmonary artery involving left upper lobe. No thrombi could be found but the pulmonary vasculature showed peripheral pruning. These findings were suggestive of chronic thromboembolic pulmonary hypertension (Figure 4).

In view of the clinical features, thrombocytopenia, photosensitivity, and malar rash a primary diagnosis of SLE was made. Although ANA was negative a diagnosis of SLE can still be made as will be discussed in the discussion part. The cause for DVT and pulmonary embolism was attributed to secondary APLA (secondary to SLE) in view of DVT, ACL, LA, and Beta 2 gp Ab being present. The lab results were later reproduced more than 12 weeks apart. The recurrent skin ulcers were attributed to the cutaneous vasculitis secondary to SLE. The cause for pulmonary hypertension was chronic thromboembolic hypertension. Thrombocytopenia was due to SLE related thrombotic thrombocytopenic purpura (TTP) like pathology. As patient had multiple issues, the management was difficult. Critical thrombocytopenia was managed with pulse methylprednisolone for 5 days followed by oral steroids. Platelet count did not improve. Option of intravenous immunoglobulins was discussed with the patient but affordability was an issue. So patient was given oral azathioprine and dapsone. Platelet count started to improve. The treatment for PAH was medical as the patient was not a candidate for pulmonary thromboendarterectomy because the distal smaller vessels were involved. Second option was bosentan (affordability was again an issue). Patient was started on phosphodiesterase inhibitors (Tadalafil). Oxygen therapy was continued. Diuretics were rationalized. Anticoagulants were started once platelets improved. The skin ulcers started healing and improving while patient received pulse steroids for thrombocytopenia. Following up the patient few months later INR is stabilized within 2–2.5 without any bleeding episode. Latest platelet counts are 243,000/micl. Skin ulcers have cleared markedly with a little scared tissue. Patient still has functional class III symptoms.

## 3. Discussion

SLE is an autoimmune disease that is usually chronic but can present acutely too. Young women are usually affected, but it can occur in up to 20 percent of patients who are older than 50 years. SLE can manifest in any organ system and the pathology may vary from minor to life-threatening [1]. There can be a preclinical phase in which the patient may be apparently normal and tests positive for autoantibodies. This is followed by a clinically overt autoimmune phase in which the clinical phenotype of the disease starts to manifest. As the disease progresses there may be flares of active disease and remissions leading to cumulative damage of the organ systems affected [2]. This discussion is focused chiefly on the presentation features of the patient described.

*SLE and Thrombocytopenia*. Thrombocytopenia can be due to multiple reasons in SLE. The prevalence ranges from 7% to 30%. This is mostly due to peripheral destruction of platelets [3]. A TTP-like syndrome may occur in SLE as was present in our patient. There can be a microangiopathic hemolytic anemia with or without the other features of TTP like fever, thrombocytopenia, kidney involvement, and neurologic symptoms. Schistocytes in the peripheral blood smear and increased lactate dehydrogenase levels are strong pointers toward this disorder. In a patient with generalized lupus activity it is referred to as TTP-like syndrome. Immunosuppressive therapy is used for treatment. In the absence of generalized lupus activity it may be referred to as bonafide TTP. A similar syndrome can also occur in the presence of antiphospholipid antibodies [2]. Serum platelet-binding IgG and platelet-associated IgG have been found to be increased in SLE with thrombocytopenia. These antibodies can also be found in SLE patients who do not have thrombocytopenia thus making it not very specific [3–5]. Thrombocytopenia is an independent risk factor for increased mortality in SLE [6]. In a retrospective study of 632 patients with SLE it was found that thrombocytopenia is not directly associated with end organ damage and mortality but defines a subgroup of patients with higher morbidity and is thus a major complication of SLE, affecting overall prognosis [7]. Sultan et al. did a study that associated thrombocytopenia with disease activity in other organs [8]. Mild thrombocytopenia may require only observation. Corticosteroids are the treatment of choice for the initial management of more severe cases [9]. Treatment is considered if bleeding or severe bruising are present, or with platelet counts <50 × 109/L [10]. Azathioprine is introduced as a steroid-sparing agent [11]. Cyclosporine is also used but should be avoided in case of kidney disease [12]. Intravenous immunoglobulins (IVIG) can be very effective in some patients with lupus-associated thrombocytopenia. IVIG in SLE act by downregulation of autoantibody production, neutralization of pathogenic autoantibodies by anti-idiotypic antibodies, inhibition of complement-mediated damage, modulation of cytokine production, induction of apoptosis in lymphocytes and monocytes, and modulation of both B- and T-lymphocyte function [13, 14]. Relatively toxic drugs like cyclophosphamide may be used in severe thrombocytopenia refractory to other less toxic drugs [15]. Other drugs used include methotrexate, romiplostim, and mycophenolate mofetil. Rituximab and stem cell transplantation may be considered in refractory cases [16]. If treatment of thrombocytopenia with steroids or other drugs is unsuccessful, splenectomy may be considered [17]. The role of splenectomy has been controversial in SLE. One of the largest series of thrombocytopenic patients with SLE who have undergone splenectomy concluded that splenectomy should be considered safe and efficacious for thrombocytopenia associated with SLE [17]. However, one older study has suggested a poor outcome [18].

*SLE and APLA*. Antiphospholipid syndrome (APS) is an autoimmune disorder characterized by thromboembolic events or fetal deaths with the presence of antiphospholipid antibodies (APLAs) [19]. APLA can be primary or secondary. Secondary APS is most commonly associated with SLE. The prevalence of APLAs in SLE is reported to be 30% to 50% [20]. As the APLAs may be transiently positive, in order to be considered a part of APS it needs to be positive 12 weeks apart as per the Sapparo criteria. The lupus anticoagulant is an independent risk factor for acute myocardial infarction and ischemic stroke [21]. The treatment for thrombotic events and its prevention center on anticoagulation. The duration of treatment differs based on the patient characteristics and an individualized decision should be taken. Unprovoked thrombotic events, arterial events, or presence of APLA support prolonged anticoagulation.

*SLE and Skin Ulcers*. Kidney is the most common organ to be affected by SLE followed by skin [22]. The 3 major types of involvement of skin in SLE are chronic cutaneous lupus erythematosus (LE), subacute cutaneous LE, and systemic or acute cutaneous LE [23]. Cutaneous manifestations are varied and include malar rash, discoid LE (DLE), photosensitivity, mucosal DLE, subacute cutaneous lupus erythematosus, alopecia, lupus panniculitis/lupus profundus, lichenoid DLE, small vessel cutaneous leukocytoclastic vasculitis secondary to LE, secondary atrophie blanche, periungual telangiectasias, livedo reticularis, Raynaud's phenomenon, and bullous lesions (BSLE) [24]. Of these, the ones which can present as cutaneous ulcers include lupus profundus and cutaneous vasculitis. Lupus profundus presents as deep brawny indurations or subcutaneous nodule. The overlying skin may be erythematous, atrophic, and ulcerated and may leave a depressed scar. The most common sites of involvement are the lateral aspects of the arms and shoulders, thighs, buttocks, trunk, breast, face, and scalp. Cutaneous vasculitis presents in a number of morphological variants including ulcers. Other lesions which it can manifest by are punctate lesions, palpable purpura, urticaria, papules, erythematosus plaques or macules, and erythema with necrosis. The most common form of vasculitis is small vessel vasculitis.

*SLE and ANA*. ANA positivity is one of the criteria in the American College of Rheumatology's criteria for the classification of SLE which is primarily used as a research tool [23]. The description of ANA-negative lupus was first raised by Koller et al. in 1976. They described five patients who were ANA-negative but had clinical features consistent with SLE [25]. Reports of ANA-negative SLE have decreased markedly in recent years probably because of the use of better substrates in ANA testing. Previously a variety of less efficient substrates were used like rat liver, mouse liver, human spleen, human prostate cell, and human granulocytes. The introduction of Hep-2 cells (a rapidly dividing human epithelial cell line) as a routine substrate for ANA determination has led to a well standardized assay with a marked increase in sensitivity [26]. Apart from antigen deficiency in the testing substrate other causes for ANA being negative in SLE include concurrent immunosuppressive treatment and persistent profound proteinuria with associated renal loss of immunoglobulins. ANA-negative lupus appeared to be a genuine and more common phenomenon a few decades ago. The current and latest evidence from the literature and experience in large centers suggests that true ANA-negative lupus is an extremely rare event [27]. In our case the substrate used for testing was Hep 2 cell based; the patient was not on immunosuppression when the testing was done and had no proteinuria. Also ANA has been repeated several months later and is negative. So our case represents a true ANA negative SLE.

*SLE and Pulmonary Arterial Hypertension (PAH)*. The prevalence of PAH in SLE is around 0.5% to 17.5%. The pathophysiology of PAH may involve multiple mechanisms. It may be due to vasculitis,* in situ* thrombosis, or interstitial pulmonary fibrosis which increases pulmonary vascular resistance. PAH is defined as an increase in mean pulmonary arterial pressure ≥25 mmHg at rest, pulmonary artery wedge pressure, or left ventricular end diastolic pressure ≤15 mmHg and increased pulmonary vascular resistance [28, 29]. The various inflammatory and autoimmune mechanisms in SLE can lead to endothelial and smooth muscle proliferation causing damage to the pulmonary vasculature and leading to PAH. Studies have shown an imbalance between vasoconstrictors and vasodilators in the pulmonary vasculature. Vascular pathologic findings in patients with SLE associated PAH include plexiform lesions, muscular hypertrophy, and intimal proliferation [30]. Chronic thromboembolic pulmonary hypertension (CTEPH) is a pulmonary vascular disease due to chronic obstruction of major pulmonary arteries. It is one of the causes of pulmonary artery hypertension. Main features of CTEPH are a nonhomogeneous distribution of disease in segments of the pulmonary vascular tree and its association with venous thromboembolism [31]. In our case the PAH was chiefly caused by CTEPH but we feel that the underlying SLE also contributed independently to it.
